# Association of Proton Pump Inhibitor and Infection and Major Adverse Clinical Events in Patients With ST-Elevation Myocardial Infarction: A Propensity Score Matching Analysis

**DOI:** 10.3389/fmed.2022.882341

**Published:** 2022-05-04

**Authors:** Yuan-Hui Liu, Zhi-Yuan Cao, Yi-Ning Dai, Li-Huan Zeng, Ye-Shen Zhang, Hua-Lin Fan, Chong-Yang Duan, Ning Tan, Peng-Cheng He

**Affiliations:** ^1^Department of Cardiology, Guangdong Cardiovascular Institute, Guangdong Provincial People’s Hospital, Guangdong Academy of Medical Sciences, Guangzhou, China; ^2^Department of Biostatistics, School of Public Health, Southern Medical University, Guangzhou, China

**Keywords:** proton pump inhibitor, infection, myocardial infarction, percutaneous coronary intervention, mortality

## Abstract

**Background:**

Infections are not common but important in patients with acute myocardial infarction, and are associated with worse outcomes. Infection was proved to be associated with the use of proton pump inhibitor (PPI) in several cohorts. It remains unclear whether PPI usage affects infection in patients with acute myocardial infarction.

**Methods:**

We consecutively enrolled patients with ST-elevation myocardial infarction (STEMI) undergoing percutaneous coronary intervention (PCI) from January 2010 to June 2018. All patients were divided into the PPI group and non-PPI group according to whether the PPI was used. The primary endpoint was the development of infection during hospitalization.

**Results:**

A total of 3027 patients were finally enrolled, with a mean age of 62.2 ± 12.6 years. 310 (10.2%) patients were developed infection during hospitalization. Baseline characteristics were similar between the PPI and non-PPI groups (n = 584 for each group) after propensity score analysis. PPI usage was significantly associated with infection based on the propensity score matching analysis (adjusted OR = 1.62, 95% CI = 1.02-2.57, *P* = 0.041). Comparing to patients with non-PPI usage, PPI administration was positively associated with higher risk of in-hospital all-cause mortality (adjusted OR = 3.25, 95% CI = 1.06-9.97, *P* = 0.039) and in-hospital major adverse clinical events (adjusted OR = 3.71, 95% CI = 1.61-8.56, *P* = 0.002). Subgroup analysis demonstrated that the impact of PPI on infection was not significantly different among patients with or without diabetes and patients with age ≥65 years or age <65 years.

**Conclusion:**

PPI usage was related to a higher incidence of infection during hospitalization, in-hospital all-cause mortality, and in-hospital major adverse clinical events (MACE) in STEMI patients.

## Introduction

Infections during hospitalization can prolong the length of intensive care unit stay and increase short-term mortality as high as tenfold for patients with acute myocardial infarction (AMI) (1–3). Standardized antibiotic therapy can be used to control an infection, but it is unlikely to reverse the worsened condition the infection typically causes. Therefore, attention should be paid to the strategies for reducing infection risk.

Proton pump inhibitors (PPIs) are routinely used to prevent gastrointestinal bleeding (4–6). The suggestions from international guidelines are diverse regarding the use of PPI treatment in post-AMI patients who receive dual antiplatelet therapy (DAPT), and the possible benefits and concerns should be carefully weighed (7–9). A large-scale study of 46301 AMI patients with DAPT found that the clinical benefits of PPI treatment were limited, even when used in accordance with the European Cardiology Society (ESC) guidelines risk stratification strategy (10) suggesting caution is warranted in prescribing PPIs. In addition, a major concern is that PPI usage may be associated with a high risk of pulmonary infection in patients with stroke (11) or critical illness (5). However, such effect was not proven in patients with cardiovascular disease. Furthermore, it remains unknown whether PPI use is associated with infection in AMI patients with a greater risk of infection. We aimed to explore the relationship between PPI use and infection in patients with ST-elevation myocardial infarction (STEMI) undergoing percutaneous coronary intervention (PCI).

## Materials and Methods

### Population

**FIGURE 1:**
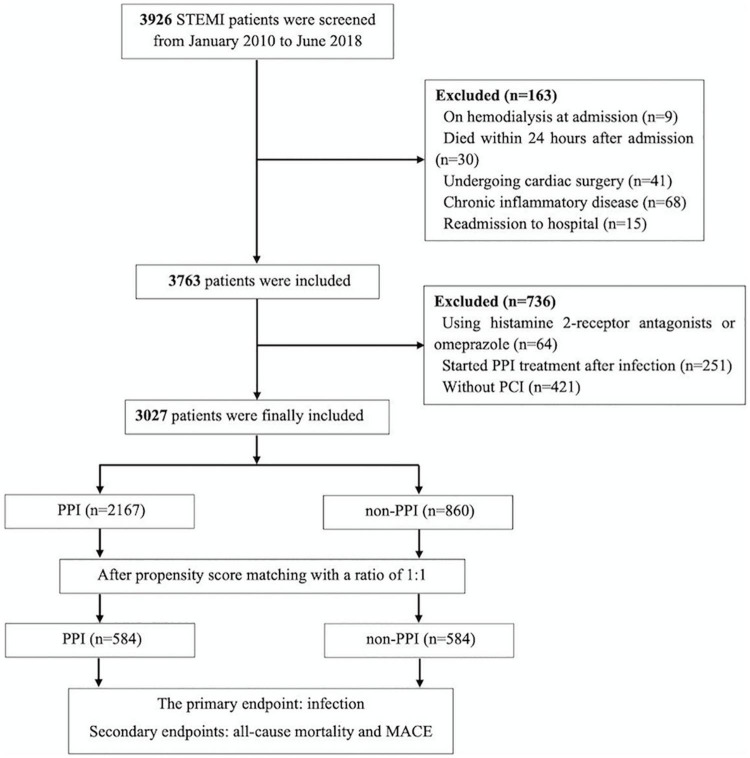
The study flow diagram.

Patients with a primary diagnosis of STEMI and admitted for undergoing PCI in Guangdong Provincial People’s Hospital from January 2010 to June 2018 were enrolled. The current updated 2017 ESC guideline was used for STEMI diagnosis (12). The exclusion criteria were on hemodialysis at admission, undergoing cardiac surgery, chronic inflammatory disease, readmission to hospital, using histamine 2-receptor antagonists or omeprazole *(not recommended in patients who received clopidogrel)*, starting PPI treatment after infection and without PCI. The study flow is shown in Figure 1. This study was conducted based on the Code of Ethics of the 1964 Declaration of Helsinki and its later amendments. The study protocol was approved by the Guangdong Provincial People’s Hospital Ethics Committee (No.GDREC2016378H), and the written informed consents were acquired.

### Baseline Clinical Data

Baseline blood samples were collected within 24 h after admission. Laboratory examination evaluated troponin I/T, blood lipids, electrolytes, serum creatinine, and other conventional parameters. A Chest X-ray was routinely performed at admission and the computed tomography scan was conducted at the physician’s discretion. The infection indicators, including culture (blood, sputum, urine, or wound), were measured when necessary. An ultrasonic cardiogram was performed during hospitalization. Coronary angiogram and PCI were performed by the cardiologists in accordance with standard interventional techniques (13).

Whether patients were administrated with PPI or not was decided at the discretion of cardiologists based on patients’ condition, and the PPIs were given by oral. Patients were separated into two groups (PPI group: received PPI within one week before admission or starting at admission until discharge; non-PPI group: did not receive PPI within one week before admission and during hospitalization). The dose and type of PPI and other medication, including antiplatelet agents, statins, angiotensin-converting enzyme inhibitors/angiotensin receptor blockers, and beta-blockers, were prescribed at the discretion of a cardiologist.

### Endpoints and Definition

The primary endpoint was infection during hospitalization, defined as infection requiring antibiotics (reflecting the clinical influence of infection compatible with the necessity for additional treatment) (14). Whether the patients needed an antibiotic was decided by the physicians, based on the sign, symptoms, and laboratory examination (such as white blood cells, C-reactive protein, and procalcitonin et al.) suggesting infection. The investigator identified infection events based on the medical record of antibiotic use and infectious features. The definition of infection was also determined in medical records at discharge with infection ICD-10 codes. Infections were classified into pulmonary infections, urinary infections, or other infections (such as abdominal sepsis, primary bacteremia, and unidentified primary infection site). Infections diagnosed within the first 72 h of hospital admission were considered as community-acquired infections, and infections diagnosed after 72 h were considered as hospital-acquired infections (15). Secondary endpoints were considered to be in-hospital all-cause mortality and in-hospital major adverse clinical events (MACE) including in-hospital all-cause mortality, stroke, recurrence of myocardial infarction, or repeated revascularization.

### Statistical Analysis

Continuous data are shown as mean ± SD and categorical data are expressed as percentages. The Student’s *t* test or analysis of variance was chosen to compare between groups when the data were normally distributed. The Wilcoxon rank-sum test was selected for comparative analysis. A multivariable logistic regression model was performed to identify the risk factors of infection, in-hospital all-cause mortality, and in-hospital MACE. To evaluate the impact of PPI on clinical outcomes, two different methods were used to adjust or balance baseline confounding factors: (1) multivariable analysis and (2) propensity score analysis. Potential confounders that were significant in the univariate analysis or that were clinically important were included in the multivariable logistic models. The following variables were ultimately included for infection: PPI use, age, sex, diabetes, anemia, peptic ulcers, hypertension, smoking, chronic obstructive pulmonary disease, prior myocardial infarction, prior stroke, white blood cell count, serum albumin levels, estimated glomerular filtration rate, DAPT, and femoral arterial access during PCI. The odds ratios (ORs) and 95% confidence intervals (CIs) were also calculated.

Three propensity score methods were conducted to test the robustness of the results: matching on the propensity score (caliper matching method of 1:1 without replacement was used with caliper set as 0.25 standard deviations of the propensity scores), covariate adjustment using the propensity score, and stratification on the quintiles of the propensity score. The propensity score model development was done using the following steps sequentially. First, the factors were all included in the logistic regression model to predict the usage of PPI. Second, for each continuous variable, the model in step one was compared with a model that incorporated the restricted cubic spline function with three knots for all continuous variables. Non-significant cubic splines were excluded. Third, we examined potential interactions between predictor variables by stepwise logistic regression (entry significant level = 0.2 and stay significant level = 0.05). Due to a large number of potential interactions, only two-way interaction was considered. Interactions that were found to be significant were then retained for subsequent steps. Fourth, we then developed a full model including all main effects, cubic-spline representations for continuous variables, and interactions that were identified in the second and third steps. This final model was then used to estimate the propensity scores. For propensity score matching analysis, patients were matched in a 1:1 (PPI group vs. non-PPI group) ratio, and a standardized difference of less than 10% was used to indicate a negligible difference in the covariates between the groups. All factors listed in Table 1 except serum creatinine [instead of eGFR (estimated glomerular filtration rate)], anemia (instead of hemoglobin), left ventricular ejection fraction, period of antibiotics, and length of hospital stay, and HbA1c were considered in the propensity score model development. For propensity score adjustment analysis, logistic regression by adjustment of only the propensity score was used to test the association of PPIs with the risk of in-hospital clinical outcomes. In the stratification analysis, patients were grouped into quintiles based on the propensity score. We also performed the analysis based on the subgroup of age ≥ 65 years, diabetes, hypertension, smoking, and eGFR < 60 mL/min/1.73 m^2^ to evaluate the role of PPI on infection. A *P* value less than 0.05 was considered statistically significant. The analyses were performed using SAS version 9.4 (SAS Institute, Cary, NC).

**TABLE 1:** Differences in baseline characteristics between patients with and without proton pump inhibitor treatment.

Variables	All patients			Propensity-matched patients			Standard difference (%)
	PPI (*n* = 2167)	non-PPI (*n* = 860)	*P*-value	PPI (*n* = 584)	non-PPI (n = 584)	*P*-value	
Age							
Age > 65 year, n (%)	971 (44.8%)	291 (33.8%)	< 0.001	206 (35.3%)	204 (34.9%)	0.902	NA
Mean (SD), year	62.40 ± 12.27	59.23 ± 11.82	< 0.001	59.85 ± 12.41	59.51 ± 11.57	0.631	2.81
Male, n (%)	1760 (81.2%)	735 (85.5%)	0.006	496 (84.9%)	495 (84.8%)	0.935	0.48
Comorbidities							
Hypertension, n (%)	1104 (50.9%)	420 (48.8%)	0.295	285 (48.8%)	276 (47.3%)	0.598	3.08
Diabetes, n (%)	583 (26.9%)	232 (27.0%)	0.967	157 (26.9%)	154 (26.4%)	0.843	1.16
Hyperlipaemia, n (%)	258 (11.9%)	111 (12.9%)	0.448	74 (12.7%)	71 (12.2%)	0.790	1.56
Smoking, n (%)	889 (41.0%)	385 (44.8%)	0.057	258 (44.2%)	258 (44.2%)	1.000	0.00
COPD, n (%)	37 (1.7%)	17 (2.0%)	0.614	11 (1.9%)	9 (1.5%)	0.652	2.64
Chronic gastritis, n (%)	63 (2.9%)	6 (0.8%)	0.001	3 (0.5%)	4 (0.7%)	0.705	−2.22
Peptic ulcer	58 (2.7%)	1 (0.1%)	< 0.001	0 (0.0%)	1 (0.2%)	0.317	−5.86
Anemia	722 (33.5%)	248 (28.9%)	0.015	184 (31.5%)	151 (25.9%)	0.033	NA
Prior myocardial infarction, n (%)	589 (27.2%)	230 (26.7%)	0.807	112 (19.2%)	106 (18.2%)	0.652	−2.64
Prior PCI, n (%)	286 (13.2%)	106 (12.3%)	0.519	44 (7.5%)	44 (7.5%)	1.000	0.00
Prior Stroke, n (%)	151 (7.0%)	37 (4.3%)	0.006	30 (5.1%)	26 (4.5%)	0.584	3.21
Atrial fibrillation, n (%)	68 (3.1%)	20 (2.3%)	0.230	19 (3.3%)	12 (2.1%)	0.203	7.46
SBP (mmHg)	122.19 ± 21.95	123.28 ± 20.70	0.200	123.30 ± 19.36	123.65 ± 20.55	0.765	−1.75
DBP (mmHg)	73.85 ± 13.63	74.64 ± 12.31	0.123	74.78 ± 13.33	74.75 ± 12.23	0.969	0.23
Killip class ≥ II, n (%)	623 (28.8%)	198 (23.0%)	0.001	128 (21.9%)	125 (21.4%)	0.831	–2.23
Examination							
White blood cell (10^9^/L)	11.51 ± 3.87	11.25 ± 3.72	0.088	11.48 ± 3.77	11.50 ± 3.67	0.915	–0.62
Total cholesterol (mmol/L)	4.87 ± 1.22	4.91 ± 1.26	0.402	4.91 ± 1.24	4.96 ± 1.22	0.486	–4.08
LDL-C (mmol/L)	3.18 ± 1.01	3.20 ± 1.03	0.538	3.21 ± 1.01	3.22 ± 1.05	0.857	–1.05
HbA1c (%)	6.10 (5.70∼6.90)	6.10 (5.70∼7.08)	0.094	6.00 (5.60∼6.90)	6.10 (5.70∼7.00)	0.099	NA
Hemoglobin (g/L)	133.58 ± 19.89	133.84 ± 23.20	0.770	134.69 ± 18.99	135.31 ± 21.36	0.601	–3.06
Serum albumin (g/L)	34.64 ± 4.26	34.75 ± 4.37	0.542	34.82 ± 3.78	34.84 ± 3.80	0.908	–0.67
eGFR (mL/min/1.73m^2^)	82.17 ± 29.92	87.56 ± 29.51	< 0.001	88.62 ± 29.71	88.62 ± 29.14	0.999	–0.01
Serum creatinine (mg/dL)	1.18 ± 1.00	1.07 ± 0.57	< 0.001	1.06 ± 0.58	1.04 ± 0.52	0.621	NA
LVEF (%)	51.80 ± 11.19	53.34 ± 11.14	0.001	51.99 ± 10.68	53.50 ± 11.13	0.023	NA
Medication							
Aspirin, n (%)	2133 (98.4%)	852 (99.1%)	0.331	574 (99.5%)	575 (99.7%)	0.654	–4.79
Clopidogrel, n (%)	2087 (96.4%)	818 (95.1%)	0.103	580 (99.3%)	582 (99.7%)	0.413	0.00
Glycoprotein IIb/IIIa inhibitors	861 (39.8%)	304 (35.6%)	0.031	231 (39.6%)	237 (40.6%)	0.720	1.52
Warfarin, n (%)	27 (1.2%)	12 (1.4%)	0.744	7 (1.2%)	7 (1.2%)	1.000	0.00
Statins, n (%)	2122 (98.0%)	841 (97.8%)	0.696	574 (98.3%)	577 (98.8%)	0.464	–4.29
ACEI/ARB, n (%)	1758 (81.1%)	743 (86.4%)	< 0.001	516 (88.4%)	518 (88.7%)	0.854	–1.07
CCB, n (%)	209 (9.6%)	81 (9.4%)	0.849	40 (6.8%)	48 (8.2%)	0.375	–5.19
Beta-blockers, n (%)	1800 (83.1%)	725 (84.3%)	0.438	489 (83.7%)	496 (84.9%)	0.573	–3.30
NSAID_S_, n (%)	28 (1.3%)	9 (1.2%)	0.840	8 (1.4%)	7 (1.2%)	0.795	1.52
Insulin, n (%)	306 (14.1%)	117 (13.6%)	0.705	82 (14.0%)	82 (14.0%)	1.000	0.00
Procedures for PCI							
Radial access, n (%)	1848 (85.4%)	755 (87.9%)	0.069	513 (87.8%)	517 (88.5%)	0.717	–2.12
Stents, No.	1.52 ± 2.50	1.48 ± 0.83	0.476	1.43 ± 0.79	1.46 ± 0.79	0.529	–3.69
Contrast volume ≥ 100 ml, n (%)	1625 (79.3%)	657 (80.1%)	0.641	464 (79.5%)	475 (81.3%)	0.418	–0.16
Multi-lesion, n (%)	1577 (72.8%)	605 (70.3%)	0.180	396 (67.8%)	397 (68.0%)	0.950	–0.37
Period of antibiotics	7.00 (5.00∼11.50)	7.00 (4.00∼12.00)	0.809				NA
Length of hospital stay, median (Q25∼Q75)	7.00 (5.00∼9.00)	6.00 (5.00∼7.75)	< 0.001	6.00 (5.00∼8.00)	6.00 (5.00∼8.00)	0.030	NA

*COPD: chronic obstructive pulmonary disease; PCI: percutaneous coronary intervention; SBP: systolic blood pressure; DBP: diastolic blood pressure; LDL-C: low-density lipoprotein cholesterol; HbA1c: hemoglobin A1c; eGFR: estimated glomerular filtration rate; LVEF: left ventricular ejection fraction; ACEI/ARB: angiotensin-converting enzyme inhibitors/angiotensin receptor blocker; CCB, calcium channel blockers; NSAIDs: Non-steroidal anti-inflammatory drugs.*

For sample size analysis, we firstly applied the rule of thumb. Events per variable should be 10 or greater. In our database, 310 patients developed an infection. It means we can adjust about 30 risk factors, and we thought the sample size was relatively enough. Furthermore, the sample size was also evaluated based on the logistic regression. A sample size of 3018 observations (of which 70% are in the non-PPI group and 30% are in the PPI group) achieved 80% power at a 0.05 significance level to detect an odds ratio of 1.5 with the R-Squared of the PPI group with other covariables set to be 0.30.

## Results

### Baseline Data

A total of 3,027 patients (17.6% female) were finally enrolled, and the mean age was 62.2 ± 12.6 years. The participants were separated into a PPI group (*n* = 2167) and a non-PPI group (*n* = 860). Patients treated with PPI are more likely to be older (44.8 vs. 33.8%, *P* < 0.001), be female (18.8 vs. 14.5%, *P* = 0.006), have a history of anemia (33.5 vs. 28.9%, *P* = 0.015), stroke (7.0 vs. 4.3%, *P* = 0.006), chronic gastritis (2.9 vs. 0.8%, *P* = 0.001), and peptic ulcer (2.7 vs. 0.1%, *P* < 0.001) than those without PPI usage. The percentage of hypertension, diabetes, hyperlipaemia, smoking, chronic obstructive pulmonary disease, prior myocardial infarction, and atrial fibrillation were similar between the two groups. Patients treated with PPI have lower level of left ventricular ejection fraction (51.80 ± 11.19 versus 53.34 ± 11.14, *P* = 0.001), eGFR (82.17 ± 29.92 mL/min/1.73 m^2^ versus 87.56 ± 29.51 mL/min/1.73 m^2^, *P* < 0.001), and higher level of serum creatinine (1.18 ± 1.00 mg/dL versus 1.07 ± 0.57 mg/dL, *P* < 0.001). The levels of white blood cells, total cholesterol, hemoglobin, and serum albumin were not significantly different between the two groups. Besides, patients treated with PPI had a higher rate of Killip class ≥ II and more use of glycoprotein IIb/IIIa inhibitors, but less use of angiotensin-converting enzyme inhibitors/angiotensin receptor blockers than those without PPI treatment. In-hospital other medications administrated, and PCI procedures were similar between the groups. In addition, the PPI group had a longer duration of hospital stay (7.00 days versus 6.00 days, *P* < 0.001) (Table 1).

### Proton Pump Inhibitor Treatment for Infection and Other Clinical Outcomes

A total of 310 (10.2%) patients developed an infection during hospitalization. The most common infection was pulmonary infection (70.0%), followed by urinary tract infection (15.5%). Patients with PPI treatment had significantly higher rates of infection than those without PPI treatment (11.5% vs. 7.0%, *P* < 0.001), including hospital-acquired infections (32.8% vs. 21.7%). However, the infection type was similar between the patients with and without PPI treatment (*P* = 0.390). The percentage of ventilator use was higher in patients with PPI treatment than in those without PPI treatment (4.8% versus 2.4%, *P* = 0.003). In addition, patients in the PPI group had a higher incidence of in-hospital all-cause mortality (3.9% vs. 1.5%, *P* < 0.001) and in-hospital MACE (5.8% vs. 2.1%, *P* < 0.001), compared with the patients in the non-PPI group.

Proton pump inhibitor use was significantly associated with the development of infection (adjusted OR = 1.76, 95% CI = 1.21–2.57, *P* = 0.003) and pulmonary infection, after adjusting for the confounding variables using multivariable logistic regression analysis (Table 2). Similar results were found for the incidence of in-hospital all-cause mortality and in-hospital MACE (Table 2). In addition, comparing to patients without PPI usage, patients with PPI usage were associated with a relatively higher risk of in-hospital all-cause mortality (OR = 1.89, 95% CI = 0.92–3.87, *P* = 0.082), and higher risk of in-hospital MACE (OR = 2.10, 95% CI = 1.18–3.73, *P* = 0.011) when further adjusting infection (Table 3).

**TABLE 2:** Multivariable analysis of proton pump inhibitor for in-hospital adverse events.

Variables	Multivariable analysis		
	OR	95% CI	*P* value
Infection*	1.76	1.21 - 2.57	0.003
Pulmonary infection^†^	1.60	1.08 - 2.39	0.020
All-cause mortality‡	2.12	1.13 - 3.98	0.019
MACE^‡^	2.42	1.43 - 4.08	0.001

MACE: major adverse clinical events. *Adjusted: age, sex, diabetes, anemia, peptic ulcer, hypertension, smoking, chronic obstructive pulmonary disease, prior myocardial infarction, prior stroke, white blood cell, serum albumin, estimated glomerular filtration rate, dual antiplatelet therapy, femoral access.

*^†^Adjusted: age, sex, anemia, hypertension, smoking, chronic obstructive pulmonary disease, prior myocardial infarction, prior stroke, white blood cell, serum albumin, estimated glomerular filtration rate, femoral access; ^‡^Adjusted: age, sex, diabetes, anemia, hypertension, smoking, chronic obstructive pulmonary disease, prior myocardial infarction, prior stroke, estimated glomerular filtration rate, femoral access.*

**TABLE 3:** Multivariable analysis of in-hospital all-cause mortality and MACE.

Variables	In-hospital all-cause mortality			In-hospital MACE		
	OR	95% CI	*P* value	OR	95% CI	*P* value
Proton pump inhibitor	1.89	0.92 - 3.87	0.082	2.10	1.18 - 3.73	0.011
Infection	4.94	2.92 - 8.34	0.000	4.87	3.16 - 7.49	0.000
Age	1.03	1.01 - 1.05	0.017	1.01	0.99 - 1.03	0.345
Sex	1.08	0.57 - 2.03	0.811	1.18	0.72 - 1.96	0.509
Diabetes	0.88	0.51 - 1.54	0.662	0.86	0.55 - 1.34	0.500
Hypertension	0.78	0.46 - 1.32	0.350	1.20	0.78 - 1.84	0.396
Prior myocardial infarction	0.31	0.14 - 0.71	0.006	0.47	0.26 - 0.83	0.009
Prior stroke	1.56	0.77 - 3.14	0.217	1.57	0.87 - 2.83	0.132
LVEF	3.00	1.79 - 5.02	0.000	1.90	1.22 - 2.95	0.005
Femoral access	2.51	1.49 - 4.23	0.001	1.71	1.10 - 2.66	0.018

*LVEF: left ventricular ejection fraction.*

### Propensity Score Analyses

**FIGURE 2:**
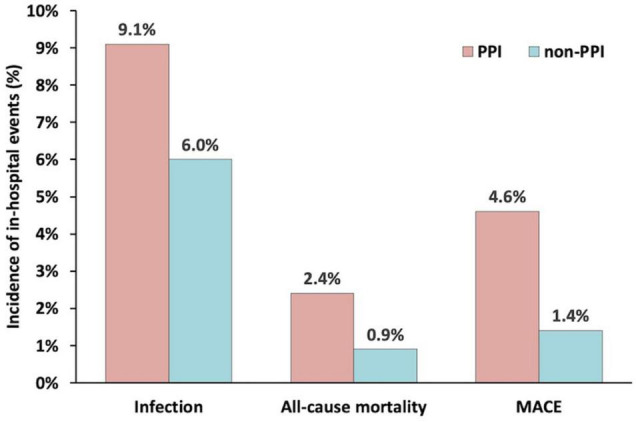
The incidence of infection, all-cause mortality, and major adverse clinical events during hospitalization in the propensity score matched groups.

Patients treated with and without PPI were then matched 1:1 to create a final cohort of 584 patients per group. The differences in baseline characteristics between the groups were eliminated by matching with all variables of a standardized difference of <10% (Table 1). After matching, the percentage of ventilator use was similar between the two groups (1.2 vs. 2.4%, *P* = 0.123). However, the rate of infection in the PPI group was still higher than that of the non-PPI group (9.1 vs. 6.0%, *P* = 0.046) (Figure 2). Similar results were found about the risk of in-hospital all-cause mortality (2.4 vs. 0.9%, *P* = 0.037) and in-hospital MACE (4.6 vs. 1.4%, *P* = 0.001).

PPI usage was significantly related to infection based on the propensity score matching analysis (adjusted OR = 1.62, 95% CI = 1.02–2.57, *P* = 0.041), propensity score adjustment analysis (adjusted OR = 1.52, 95% CI = 1.06–2.18, *P* = 0.023), and stratification analysis (adjusted OR = 1.53, 95% CI = 1.06–2.20, *P* = 0.022). Both propensity score analyses demonstrated that PPI administration was positively associated with in-hospital all-cause mortality and in-hospital MACE (Table 4).

**TABLE 4:** Propensity score analysis of in-hospital clinical outcomes.

Outcomes	Propensity scores matching analysis (1:1)			Analysis by adjusting the propensity scores			Stratification analysis		
	OR	95% CI	*P*	OR	95% CI	*P*	OR	95% CI	*P*
Infection	1.62	1.02 - 2.57	0.041	1.52	1.06 - 2.18	0.023	1.53	1.06 - 2.20	0.022
All-cause mortality	3.25	1.06 - 9.97	0.039	2.82	1.11 - 7.16	0.029	2.97	1.15 - 7.62	0.024
MACE	3.71	1.61 - 8.56	0.002	3.01	1.50 - 6.06	0.002	2.98	1.47 - 6.05	0.002

*MACE: major adverse clinical events.*

### Subgroup Analysis

**FIGURE 3:**
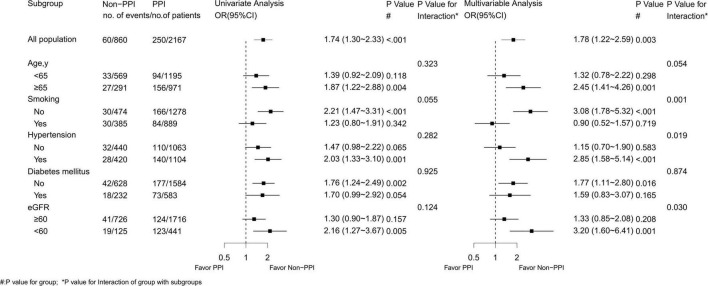
Subgroup analysis of infection.

The impact of PPI on infection was not significantly different among patients with or without diabetes and those patients with age ≥ 65 years or age < 65 years. A significant interaction of the effect of PPI on the infection has been found between patients with or without smoking, hypertension, and with eGFR > 60 mL/min/1.73 m^2^ or < 60 mL/min/1.73m^2^. Patients with a history of hypertension or with eGFR < 60 mL/min/1.73 m^2^ had a higher risk of infections than those without a history of hypertension or with eGFR > 60 mL/min/1.73 m^2^, when they were treated with PPI. However, patients treated with PPI and without smoking had a higher risk of infections than those with smoking (Figure 3).

## Discussion

The present study demonstrated that PPI treatment was related to a high risk of infection during hospitalization and other in-hospital clinical outcomes.

Growing evidence from large-scale epidemiology studies has revealed that PPI use was associated with elevated risks of both community-acquired and hospital-acquired pneumonia (16–18). Studies exploring PPI usage and pulmonary infection were based on critical illness cohorts. A meta-analysis of 72 randomized controlled trials suggested that PPI administration might increase pneumonia in clinical illness, which was supported by clinical practice guidelines in 2020 (19, 20). However, another meta-analysis indicated that prophylactic PPI administration might have no significant impact on pneumonia, the discrepancies may be due to methodologic differences or insufficient data (5). Compared to patients with AMI, critical illness patients also had high rates of infection, but were more complex with fixed diseases; infection development in the critical illness cohort might be related to the ventilators and prolonged intensive care unit stays (21, 22). Variation in the factors involved in the development of infection made these two cohorts independent of each other. Thus, the evidence from a critical illness cohort might be not suitable extrapolation to an AMI cohort.

PPI treatment has been proven to be associated with increased odds of hospital-acquired pneumonia in patients with acute stroke (11). However, it is unclear whether these results can be extrapolated to patients with AMI. In a large multicenter randomized study in patients with stable coronary or peripheral arterial disease who were given rivaroxaban or aspirin, long-term PPI usage was not associated with increased pneumonia (6). The incidence of pneumonia was only 2.8% in these patients compared to ours, likely due to the inclusion of low-risk patients, such as those with stable coronary disease. An observational study including 332 acute coronary syndrome patients found that the incidence of hospital-acquired pneumonia was not significantly different between patients who received PPI treatment and those who did not (23). First of all, the cohort sizes were small; and they included patients with high risk for gastrointestinal hemorrhage and mortality, the 30-day mortality reached 10%, which further limited the assessment of PPI for pneumonia. Second, only hospital-acquired pneumonia was evaluated that largely underestimating the pneumonia diagnosis within 48 hours. However, the study herein might be the first large-scale analysis to show that PPI therapy is significantly associated with the infection including community-and hospital-acquired infection in STEMI patients undergoing PCI. In addition, to the lecture on PPI and infectious diseases, previous studies focus on pneumonia or *Clostridium difficile* infection, our present findings extended it to the overall infection (24–27).

PPI usage associated with increasing infection is largely driven by pulmonary infection in our study. The mechanisms by which PPI usage increases pulmonary infection remain unknown, especially in patients with AMI. The rational explanation is that PPI adversely lower PH and promotes aerobic bacteria in the stomach, then alters the gut microbiome; it may increase spontaneous bacterial peritonitis or pneumonia (28). This hypothesis implies that these changes facilitate lung infection following minor aspirations, as in aspiration pneumonia (29). It is also reported that PPI might induce hypochlorhydria to damage the defense against ingested bacteria and viruses (30, 31). Additionally, PPI usage was associated with neutrophil function impairment *in vitro*, which might reduce immune activation on the bacterial challenge (32). On the other hand, PPI usage increasing MACE was partly attributed to the infection events; and may also by alleviating the activity of nitric oxide synthase (33).

The present study results support and confirm previous observational studies that PPI therapy was associated with a high risk of all-cause mortality and MACE (34–37). In a *post hoc* analysis of the PLATO (Platelet Inhibition and Patient Outcomes) trial, PPI use was independently associated with a higher risk of cardiovascular events for both clopidogrel and ticagrelor (38). Recent meta-analyses, including observational studies, also demonstrated similar results (39, 40). In contrast, randomized clinical trials did not prove that PPI treatment would worsen cardiovascular outcomes (41, 42). This might be explained by the fact that PPI use in the observational studies was a marker rather than a cause of cardiovascular complications. To date, the 2017 ESC guideline recommends PPI use to protect against gastrointestinal hemorrhage in patients who received DAPT, while the 2015 ESC regards combination PPI usage in those at higher risk of bleeding (7, 8). Additionally, the 2014 AHA/ACC suggests that PPI therapy should be considered in patients who required triple antithrombotic therapy although they were without a history of gastrointestinal hemorrhage (9). Despite the conflicting advice, for AMI patients treated with DAPT, the incidence of gastrointestinal hemorrhage (either upper gastrointestinal hemorrhage or any gastrointestinal hemorrhage) was low even in the high-risk patients (10, 43). This provides justification for withholding PPI in routine cases. While our study advances the understanding of PPI in AMI-DAPT subjects, there remains a need for ongoing risk stratification and case-by-case evaluation rather than routine PPI administration, especially in patients considered to be at high risk of infection.

The present study has a number of limitations. First, although we performed multivariable analyses and propensity score analyses to reduce the bias, undetected confounders might still exist for this single-center observational study. Second, we did not assess the effectiveness of PPIs in gastrointestinal hemorrhage prevention and compare the effect of different PPIs, modes of administration and regimens on the results. Future studies are necessary to better understand the net clinical effect of PPI usage and different PPIs on the infection. Third, our cohort was limited to STEMI patients and most of them were treated with clopidogrel. Thus, the findings cannot necessarily be applied to other cardiac patients or those treated with ticagrelor. Forth, the study lacked follow-up data on post-discharge infections. It is important to investigate this to assess the impact of PPI therapy on late occurring infections. And the study also lacked data on cardiovascular mortality. Fifth, according to our current definition of infection, we would miss those patients caused by viruses or include those patients with inappropriate antibiotics applications in clinical practice. Noticeably, some inflammatory indicators, such as C-reactive protein and procalcitonin, were not included in the current study since they were unconventional detection indexes, especially before the PCI procedure. And antibiotic type might also influence the outcome. Future research should include these variables to further evaluate their effects on outcomes.

## Conclusion

For patients with STEMI undergoing PCI, PPI usage during hospitalization was related to a higher risk of infection as well as in-hospital all-cause mortality and MACE.

## Data Availability Statement

The data used to support the findings of this study are available from the corresponding author upon request. Requests to access these datasets should be directed to P-CH, gdhpc100@126.com.

## Ethics Statement

The studies involving human participants were reviewed and approved by the Guangdong Provincial People’s Hospital Ethics Committee. The patients/participants provided their written informed consent to participate in this study.

## Author Contributions

Y-HL, NT, and P-CH: study concept and design. Z-YC, Y-HL, and Y-ND: drafting of the manuscript. C-YD and Z-YC: supervisors of the study and guarantee the study data and accuracy. All authors contributed to the acquisition, analysis, or interpretation of data, critical revision and final approval of the manuscript.

## Conflict of Interest

The authors declare that the research was conducted in the absence of any commercial or financial relationships that could be construed as a potential conflict of interest.

## Publisher’s Note

All claims expressed in this article are solely those of the authors and do not necessarily represent those of their affiliated organizations, or those of the publisher, the editors and the reviewers. Any product that may be evaluated in this article, or claim that may be made by its manufacturer, is not guaranteed or endorsed by the publisher.
